# Nursing care for parents who have experienced fetal demise: integrative review

**DOI:** 10.1590/0034-7167-2022-0811

**Published:** 2024-03-15

**Authors:** Nycarla de Araújo Bezerra, Cibelle Nayara Sena dos Santos, Adrian Thaís Cardoso Santos Gomes da Silva, Francisca Márcia Pereira Linhares, Sheila Coelho Ramalho Vasconcelos Morais

**Affiliations:** I Universidade Federal de Pernambuco. Recife, Pernambuco, Brazil

**Keywords:** Fetal Death, Bereavement, Professional Practice, Nursing Care, Review, Muerte Fetal, Aflicción, Práctica Profesional, Atención de Enfermería, Revisión, Morte Fetal, Luto, Prática Profissional, Cuidados de Enfermagem, Revisão

## Abstract

**Objectives:**

to identify scientific evidence regarding nursing care for parents who have experienced grief following fetal demise.

**Methods:**

an integrative review of original studies was conducted across six databases. The studies were classified according to the level of evidence.

**Results:**

the qualitative analysis of the nine studies comprising the sample involved thematic categories, exploring the impact of perinatal loss on families, inadequate communication by healthcare professionals, and the importance of a holistic approach in care. The role of the nurse is highlighted in making a positive contribution to the team, emphasizing participation in training and the provision of essential information.

**Final Considerations:**

grieving affects not only family dynamics but also the social environment, emphasizing the urgency of a more empathetic and comprehensive approach. Care should be holistic, going beyond technical nursing assistance, and addressing the biopsychosocial context of the parents.

## RESUMO

**Objetivos:**

identificar evidências científicas acerca do cuidado de enfermagem aos pais que vivenciaram o luto diante do óbito fetal.

**Métodos:**

revisão integrativa de estudos originais realizada em seis bases de dados e classificados quanto ao nível de evidência.

**Resultados:**

a análise qualitativa dos nove estudos que compuseram a amostra envolveu categorias temáticas, explorando o impacto da perda perinatal nas famílias, a comunicação inadequada por parte dos profissionais de saúde e a importância de uma abordagem holística na assistência. Destaca-se a importância do papel do enfermeiro na contribuição positiva para a equipe, enfatizando a participação em capacitações e o fornecimento de informações essenciais.

**Considerações Finais:**

a vivência do luto impacta não apenas a dinâmica familiar, mas o meio social, evidenciando a urgência de uma abordagem mais empática e compreensiva. O cuidado deve ocorrer de forma holística, transcendendo a assistência técnica de enfermagem, com abordagem do contexto biopsicossocial dos pais.

## RESUMEN

**Objetivos:**

identificar evidencia científica sobre el cuidado de enfermería a padres que han experimentado el duelo ante la pérdida fetal.

**Métodos:**

revisión integradora de estudios originales realizada en seis bases de datos y clasificados según el nivel de evidencia.

**Resultados:**

el análisis cualitativo de los nueve estudios que conformaron la muestra abordó categorías temáticas, explorando el impacto de la pérdida perinatal en las familias, la comunicación inadecuada por parte de los profesionales de la salud y la importancia de un enfoque holístico en la atención. Se destaca la importancia del papel del enfermero en la contribución positiva al equipo, haciendo hincapié en la participación en capacitaciones y la provisión de información esencial.

**Consideraciones Finales:**

la experiencia del duelo afecta no solo la dinámica familiar, sino también el entorno social, evidenciando la urgencia de un enfoque más empático y comprensivo. El cuidado debe ser holístico, trascendiendo la asistencia técnica de enfermería, con un enfoque en el contexto biopsicosocial de los padres.

## INTRODUCTION

Pregnancy is seen as synonymous with life, a moment when expectations increase with the development of the fetus, strengthening the mother/father-child bond and deepening emotional ties. This period can be interrupted when fetal demise occurs, a concrete and finite loss in which the entire symbolism of life is shattered, potentially resulting in profound and traumatic marks on parents and families experiencing grief^(1)^.

The process of death and dying in the context of childbirth, especially when unexpected, can hinder the adaptation of all involved parties, whether mothers and fathers or the professionals engaged^(2-3)^. Fetal demise can occur from the 22nd complete week of gestation or in fetuses with a weight equal to or greater than 500g or a length of at least 25 cm^(4)^.

Confirmation of fetal death can be a psychologically traumatic event for the woman and her family, with long-term effects extending beyond the emotional realm into the physical and social spheres. Therefore, qualified care during the grieving process by the healthcare team is essential for family coping, recognizing that the pain of losing a fetus is often underestimated, concealed, and overlooked in the social space^(5-7)^.

Challenges in providing humanized and holistic care to parents experiencing this moment have been a challenging practice for healthcare professionals^(3-6)^. Nursing professionals face difficulties in caring for parents experiencing fetal demise. Specific preparation is required to assist in the grieving process, involving the development of competencies and skills for effective communication. The adequate training of these professionals is a fundamental axis for holistic care^(6)^.

Guidance for a nursing care plan that meets the needs of both the mother and father requires qualified listening during labor, delivery, and the postpartum period^(7)^. Weaknesses have been identified in the care provided to these parents, related to the services offered in healthcare. In addition to dissatisfaction with underestimation, trivializations, and iatrogenesis, problems related to the absence of a physical structure and adequate human resources to address not only technical but also the psychosocial needs of parents experiencing loss and grief are observed^(5-7)^.

Despite publications on women’s health and obstetrics, attention specifically focused on parents in situations of fetal loss has not yet been a priority in public health policies. Furthermore, knowledge gaps regarding scientific production indicate the need to advance investigations that reveal the demands and support the development of care plans aimed at comprehensive and qualified assistance for these parents.

## OBJECTIVES

To identify scientific evidence regarding nursing care for parents who have experienced grief following fetal demise.

## METHODS

### Ethical Aspects

The Institutional Review Board’s approval was waived since this is a review study using publicly available data and does not involve human subjects.

### Study Design

This is an integrative review aimed at synthesizing knowledge, identifying research gaps, and suggesting new studies. The process follows a systematic and rigorous approach without a specific time frame to gather as many articles as possible on the topic. The study was conducted in six stages:

1. Identification of the theme and guiding question;
2. Establishment of inclusion and exclusion criteria;
3. Definition of information to be extracted from selected studies and characterization;
4. Evaluation of included studies;
5. Interpretation of results;
6. Presentation of the review/knowledge synthesis^(8-9)^.

### Identification of the Research Question

Initially, the PICo strategy^(10)^ was used to formulate the guiding question:

- P (Population): Parents experiencing grief;
- I (Interest): Nursing care;
- Co (Context): Fetal demise.

Thus, the research question adopted was: What is the scientific evidence regarding nursing care for parents who have experienced grief following fetal demise? Subsequently, inclusion criteria were established: Original articles, without a time limitation for searches, published in Portuguese, English, and Spanish, and related to the guiding question. As for exclusion criteria, gray literature (theses, dissertations, monographs, books, book chapters, congress abstracts, proceedings, government reports, review articles) and publications not addressing the study’s guiding question were considered.

### Identification of Relevant Studies

Data collection from the literature was conducted between August and September 2022 through searches in electronic databases/libraries: Pubmed Central (PMC), Medical Literature Analysis and Retrieval System Online (MEDLINE), Cumulative Index of Nursing and Allied Health Literature (CINAHL), *Literatura Latino Americana e do Caribe em Ciências da Saúde* (LILACS), Scopus (Elsevier), and Web of Science (WoS).

For article searches, the following Health Science Descriptors (DeCS) were used: “Grief,” “Professional Practice,” “Nursing Care,” “Fetal Death,” along with their English equivalents available in the Medical Subject Headings (MeSH). Search strategies were adapted for each database according to their specificities, using the PICo strategy, keywords, and entry terms, separated by boolean operators OR for distinction and AND for association, to integrate and direct as many studies on the topic as possible, as shown in Chart 1.

**Chart 1:** Search strategy in databases based on the PICo strategy, Recife, Pernambuco, Brazil, 2022

Database	Search Strategy
PUBMED Central (PMC)	P AND I AND Co P ALL FIELDS “Bereavement” OR “Bereavements” OR “Maternal Mourning” AND I ALL FIELDS “Professional Practice” OR “Practice, Professional” OR “Practices, Professional” OR “Professional Practices” OR “Nursing Care”OR “Nursing, Care” OR “Nursings, Care” OR “Nursing” AND Co ALL FIELDS “Fetal Death” OR “Death, Fetal” OR “Deaths, Fetal” OR “Demise, Fetal” OR “Fetal Deaths” OR “Fetal Demise” OR “Fetal Mummification” OR “Mummification Fetal”
MEDLINE	P AND I AND Co P ALL FIELDS “Bereavement” OR “Bereavements” OR “Maternal Mourning” AND I ALL FIELDS “Professional Practice” OR “Practice, Professional” OR “Practices, Professional” OR “Professional Practices” OR “Nursing Care”OR “Nursing, Care” OR “Nursings, Care” OR “Nursing” AND Co ALL FIELDS “Fetal Death” OR “Death, Fetal” OR “Deaths, Fetal” OR “Demise, Fetal” OR “Fetal Deaths” OR “Fetal Demise” OR “Fetal Mummification” OR “Mummification Fetal”
CINAHL	P AND I AND Co P ALL FIELDS “Bereavement” OR “Bereavements” OR “Maternal Mourning” AND I ALL FIELDS “Professional Practice” OR “Practice, Professional” OR “Practices, Professional” OR “Professional Practices” OR “Nursing Care”OR “Nursing, Care” OR “Nursings, Care” OR “Nursing” AND Co ALL FIELDS “Fetal Death” OR “Death, Fetal” OR “Deaths, Fetal” OR “Demise, Fetal” OR “Fetal Deaths” OR “Fetal Demise” OR “Fetal Mummification” OR “Mummification Fetal”
SCOPUS	P AND I AND Co P ALL FIELDS “Bereavement” OR “Bereavements” OR “Maternal Mourning” AND I ALL FIELDS “Professional Practice” OR “Practice, Professional” OR “Practices, Professional” OR “Professional Practices” OR “Nursing Care”OR “Nursing, Care” OR “Nursings, Care” OR “Nursing” AND Co ALL FIELDS “Fetal Death” OR “Death, Fetal” OR “Deaths, Fetal” OR “Demise, Fetal” OR “Fetal Deaths” OR “Fetal Demise” OR “Fetal Mummification” OR “Mummification Fetal”
BVS LILACS	P AND I AND Co P ALL FIELDS “Bereavement” OR “Bereavements” OR “Maternal Mourning” AND I ALL FIELDS “Professional Practice” OR “Practice, Professional” OR “Practices, Professional” OR “Professional Practices” OR “Nursing Care”OR “Nursing, Care” OR “Nursings, Care” OR “Nursing” AND Co ALL FIELDS “Fetal Death” OR “Death, Fetal” OR “Deaths, Fetal” OR “Demise, Fetal” OR “Fetal Deaths” OR “Fetal Demise” OR “Fetal Mummification” OR “Mummification Fetal”
Web of Science	P AND I AND Co P ALL FIELDS “Bereavement” OR “Bereavements” OR “Maternal Mourning” AND I ALL FIELDS “Professional Practice” OR “Practice, Professional” OR “Practices, Professional” OR “Professional Practices” OR “Nursing Care”OR “Nursing, Care” OR “Nursings, Care” OR “Nursing” AND Co ALL FIELDS “Fetal Death” OR “Death, Fetal” OR “Deaths, Fetal” OR “Demise, Fetal” OR “Fetal Deaths” OR “Fetal Demise” OR “Fetal Mummification” OR “Mummification Fetal”

### Study Selection

After collecting scientific publications, we organized studies using the Mendeley reference manager to identify and eliminate duplicates. Each repeated study was counted only once. Subsequently, we transferred them to the online platform Rayyan QCRI, where the title and abstract underwent independent review by two researchers. Studies aligned with the study’s theme were included in the sample. Any discrepancies in selection were resolved through discussion between the two researchers to reach a final decision.

### Data Analysis

For data analysis, a full-text reading was conducted to confirm the eligibility of selected studies. We analyzed and categorized those fully or partially addressing the research question of this review. Studies not addressing this question were excluded, resulting in a final sample of nine scientific articles.

Information extraction utilized a standardized form^(11)^, capturing details such as author(s), publication year and country, objectives, outcomes, and level of evidence.

The level of evidence was evaluated following the hierarchical classification of Melnyk and Fineout-Overholt (2011)^(12)^:

- I: Evidence from a systematic review, meta-analysis, or clinical practice guidelines based on systematic reviews of randomized controlled trials;
- II: Evidence from at least one well-designed randomized controlled trial;
- III: Evidence derived from well-designed trials without randomization;
- IV: Evidence from well-designed cohort and case-control studies;
- V: Evidence from a systematic review of descriptive and qualitative studies;
- VI: Evidence from a single descriptive or qualitative study;
- VII: Evidence derived from the opinion of authorities and/or expert committee reports.

Data analysis involved content analysis of articles, starting with pre-analysis, floating evidence reading, organizing convergent information, exploring findings, and identifying thematic categories^(13)^.

## RESULTS

**Figure 1:**
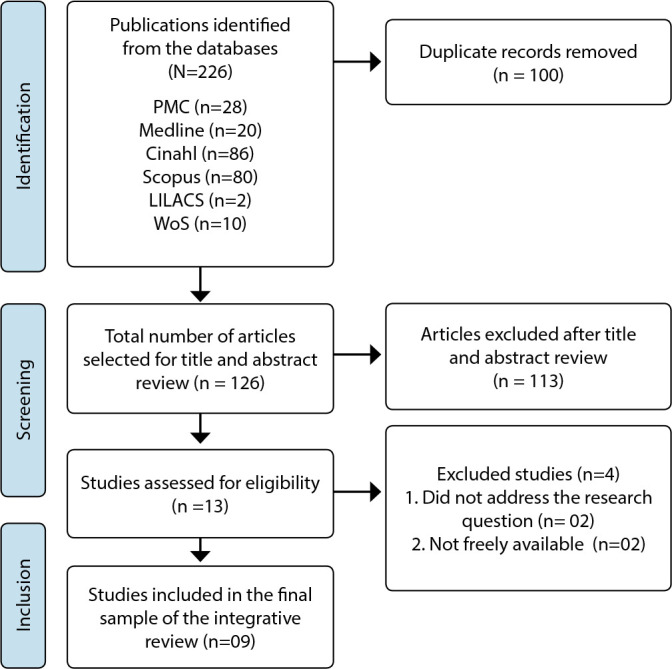
Flowchart of the selection of primary studies included in the integrative review

*Source: Page et al.^(14)^*

For presenting the search and article selection stage, we opted to use the study selection flowchart adapted from the Preferred Reporting Items for Systematic Reviews and Meta-Analyses (PRISMA). Thus, Figure 1 outlines the process undertaken for the identification, screening, eligibility, and inclusion of studies, according to the consulted databases. Initially, 226 publications were identified, of which 13 met the eligibility criteria; however, only 09 were included in the final sample.

*PMC - Pubmed Central; Medline - Medical Literature Analysis and Retrieval System Online; CINAHL - Cumulative Index to Nursing and Allied Health Literature; LILACS - Literatura Latino-Americana e do Caribe em Ciências da Saúde; Scopus and WoS - Web of Science; n - quantity.*

### Temporal Cut-off and Characteristics of Selected Studies

Concerning the temporal cut-off, the publications ranged from 2016 to 2021. Among the nine selected studies, the majority were published in 2020, totaling (n=3), followed by the years 2016, 2019, and 2021, each with two studies. Regarding the language of publication, eight were written in English, and only one in Portuguese. In terms of geographical origin, the studies were conducted in the following countries: Spain (n=3), Brazil/Canada, Kenya/Uganda, Israel, Afghanistan, China, and Ireland, each with a single publication.

Regarding the level of evidence in the studies, (n=5) articles stood out with level VI - evidence derived from a single descriptive or qualitative study, followed by (n=3) with level V - evidence presented in a systematic review, descriptive, and qualitative studies, and (n=1) article with level I - evidence resulting from a systematic review, meta-analysis, or clinical guidelines from systematic reviews of randomized controlled trials.

The sample of this study, based on the analyzed articles, is demonstrated in a synthesized form in the summary table (Chart 2).

**Chart 2:** Characterization of articles, including authors, year, country of publication, objectives, outcomes, and Level of evidence, Brazil, 2022

Study and Authors	Year/ Country	Objectives	Outcomes	Level of Evidence
E1^(15)^ Qian, J et al.	2021 China	Provide a much-needed and comprehensive overview of current programs for perinatal grief care education, thus laying the foundation for the development and optimization of educational programs while identifying needs for future research.	A comprehensive view of the current state of perinatal grief care education programs was presented, both in university and hospital settings. It was observed that the implementation of these programs has positive effects in assisting nurses and midwives in preparing for perinatal grief care.	I
E2^(16)^ Mills TA et al.	2020 Kenya / Uganda	Explore the experiences of parents in the period following the death of their baby in healthcare units in Kenya and Uganda to understand the healthcare system’s response and barriers and facilitators for effective support.	Parents were observed to describe devastating impacts and profound responses to the baby’s death. Interactions with healthcare professionals were significant influences, but deficient communication was noted. Environmental barriers and unsupportive facility practices meant that needs were often unmet.	V
E3^(17)^ Ávila MC et al.	2019 Spain	Describe and understand the experiences and perceptions of parents who have suffered perinatal death.	Parents facing perinatal loss tend to anticipate the baby’s death, often perceiving that “something was wrong with the pregnancy.” It was also evident that the shock of losing a baby and the pain of giving birth to a dead baby trigger an emotional wave in parents who feel they “had a child” and need to assign an identity to legitimize grief.	IV
E4^(18)^ Christou A et al	2021 Afghanistan	Explore the experiences of bereaved parents and healthcare professionals in postpartum care.	Inadequate and insensitive communication and practices by healthcare professionals, including avoiding or delaying the disclosure of fetal death.	IV
E5^(19)^ Kalu FA et al	2020 Ireland	Develop a validated and reliable scale for perinatal grief care trust.	Hospitals were not equipped to separate women who had stillbirths and acknowledged that psychological support would be beneficial. However, the lack of trained personnel and resource constraints hindered the provision of such support.	IV
E6^(20)^ Fernández-Sola C et al	2020 Spain	Explore, describe, and understand the impact of perinatal death on the social and family lives of parents.	The use of the Perinatal Grief Care Trust Scale will facilitate the identification of needs and educational and support strategies to promote midwives’ knowledge and skills in supporting parents who have experienced perinatal loss. The results of these assessments will provide input for areas of education and training in grief care that need improvement to enhance midwives’ perinatal support knowledge and skills in practice.	IV
E7^(21)^ Golan A; Leichtentritt DR.	2016 Israel	Examine the meaning that women who have experienced the loss of a child attribute to their loss and the lost figure.	Perinatal death affects family dynamics, including a couple’s relationship, which can be strengthened or weakened. It is important for women to be informed by professionals about the loss directly and respectfully, avoiding language that minimizes the loss and may hinder attempts to forge personal meaning for it.	V
E8^(22)^ Martínez PS et al.	2019 Spain	Explore the experiences of mothers and fathers regarding the care received during childbirth in cases of stillbirth.	Healthcare professionals and educators need to understand the impact of this loss on family dynamics, as well as on the social and work environment, paying attention not only to mothers but also to fathers and siblings.	IV
E9^(23)^ Paris GF; Montigny F; Pelloso MS.	2016 Brazil/ Canada	Investigate the association between complicated grief and sociodemographic, reproductive, mental, marital satisfaction, and professional support characteristics in women after fetal demise.	Provides insight into the process of meaning reconstruction after fetal demise. Identifies grief after this loss as a private disenfranchised grief. To create meaning for birth-related losses, one must first understand who was lost. The results of this study also indicate that coping with loss by women is a long-term process.	V

In light of the above, the critical analysis and synthesis of the selected studies were conducted qualitatively, leading to the categorization of three main themes: Fetal death and family dynamics; the Need for legitimizing grief; and Nursing care and the organizational and structural aspects of services.

## DISCUSSION

### Fetal Death and Family Dynamics

In this category, the impact of perinatal death on individual life and family dynamics was addressed. The grieving process is experienced by everyone involved and transcends each person’s personal space, affecting work and social environments^(17-20)^. The impact of fetal death reflects on the emotional, physical, and psychological dimensions of the couple and each family member^(16,18)^. The construction of this family, filled with dreams, is interrupted, and the feelings after grief are often veiled by unspoken communication and silence. This persists in grieving families and is associated with how fetal loss is experienced and represented in society, leading to a delegitimization of this grief^(20-22)^.

Regarding the impact on the work and social environments they are part of, social interaction becomes a challenge for parents when faced with other families with newborns and receiving stereotypical messages of comfort. From the studies, it was observed that these moments intensify the pain because this comfort comes directly from a family that has recently had a healthy child. Social contact is avoided by parents due to the difficulty of dealing with loss, lack of empathy, and society’s failure to recognize grief^(22-23)^.

Another aspect to consider is the emphasis on emotional dimensions when the mother and father express difficulty accepting the loss and develop anxiety and depression disorders. During the grieving process, it is important to direct attention not only to the mother but also to include the father. Studies suggest that society and healthcare services should value the feelings of these families, often minimized and invisible, making holistic care planning challenging^(16-18,20)^. It is emphasized that focusing solely on the mother weakens family care, requiring a broad view from the healthcare team so that this grieving process can be faced congruently, giving meaning to the loss and strengthening the marital bond^(20-22)^.

In this sense, the loss of a child causes an imbalance in the family nucleus. Parents need to cope with the rupture of the bond and make sense of the loss. In the experience of absence, it is necessary to restructure a new reality without the child. Although grief is considered a unique process, it depends on other factors, such as how this loss is felt, the psychological resources of the sufferer, personal history, as well as available support relationships and respect for maternal and paternal pain. This support and respect for the grief of both the mother and father are crucial^(23-24)^.

Parents express different feelings, and the repression of these feelings by society in the face of fetal death, especially by the father, where feelings of guilt are also experienced, seeking to identify what they did not do or could have done differently, where they failed^(25)^. Often, there is no moment of listening and welcoming of his pain. This reflects a cultural scenario where it is expected that this father be strong to support the woman, even losing the right to cry. It is necessary to create spaces for men to express their feelings and feel authorized to express their pain^(23-25)^.

Supportive bonds play an important role, even in preventing grief complications. The grieving process requires parents to find moments and spaces to reframe such psychic rupture, respecting each person’s process. This task requires the mobilization of subjective resources that allow the reframing of loss and the continuation of life in the face of lived experiences^(22-24)^.

### Need for Grief Legitimization

Grief is an event that requires adaptation to loss, and when this path is not valued, legitimized, or leaves gaps, it becomes even more challenging to navigate. Thus, it involves a cognitive process that includes coping, restructuring of feelings and the experience of loss, and changes in daily life due to the death of the child^(25)^.

Parents appreciated the efforts of professionals who provided empathetic farewells, allowing physical contact and participation in rituals such as cutting the umbilical cord or receiving impressions of the baby’s feet and/or placentas as memories of the birth^(15)^. According to these experiences, this facilitates the task of establishing a lasting connection with the departed child. These parents express gratitude to the teams for the moments they could be with the baby, see it, and touch it, understanding that it was a very delicate moment, but they knew they needed to say goodbye because, although not born alive, it was the expected child and would be forever^(15,19,26)^.

It is considered that each family has its mourning ritual, and the importance of respecting individual beliefs assists in elaborating and legitimizing the experienced grief. Grief resulting from fetal loss differs from grief that occurs with other deaths and presents a higher risk of complications^(16,18-19)^. This lengthy process begins with the notification of fetal death, followed by care provided during labor and extends to the couple’s home for a variable period, which can last several years^(15,18,22)^.

Fetal loss implies a paradoxical experience that does not occur in other life circumstances. The act of childbirth, linked to life, birth, and the death of the child simultaneously induces perplexity for parents who experience a sense of frustration, intense pain, and incomprehension because they were not prepared to experience the situation where life and death walk together, and one does not need to grow old to die^(16,24-25)^.

Losing a child can be considered one of the most devastating events and involves three different moments: the past, where the dream was idealized; disillusionment and sadness, which seem endless for the present moment; and doubt about the future^(22-24)^.

Although parents directly suffer from grief, it is important to note that family members and friends also feel the loss. However, studies show that, in many cases, there is silencing from these individuals, as they expect both the mother and father to expedite their grief processing. These individuals make comments that soon the mother can get pregnant again, for example, in an attempt to replace the lost child, or that they are young and can have as many children as they want. In these situations, there is a devalidation and silencing of the loss and grief process^(21,26)^.

Understanding how this processing occurs is important because it is significant to recognize the bond that exists between parents and the child. Grief can function as an emotional destabilizer for a person, as society is not ethically and appropriately prepared to deal with the feeling of loss, especially when there is an emotional bond with the departed^(17,20,27-29)^.

The suffering generated by grief related to fetal death is more intense due to the idealization built around the anticipation of a healthy birth that did not materialize. Because of this sentiment, parents are prone to developing issues such as depression, panic disorder, and anxiety, especially when the loss is recent. In this way, it is understood that this process is individual^(21,26)^.

From the studies, it was observed that the process of reframing is always unique and occurs at different times for each mother, father, couple, and family. Nevertheless, it is essential to highlight the importance of spirituality, engagement in projects related to the departed child, and the strengthening of bonds with significant individuals for coping. These factors are possible components of a restructuring of the relationship with the child who passed away^(16,18,21-28)^. Thus, based on respected experiences, these parents can experience grief in a less painful way and receive appropriate support^(18,21)^.

### Nursing Care, Organizational Aspects, and Structural Aspects of Services

The importance of preparatory care to obtain targeted nursing care for families experiencing fetal death is evident. It is crucial to understand that these family members need to go through the stages of grief with as little suffering as possible. Healthcare services need to adapt and provide a space to actively embrace the emotions and feelings of these parents so that they can find meaning in this absence^(19,24,28)^.

Teams should employ therapeutic communication, using their knowledge and skills to assist in facing the challenges of patients and their families. Communication, health, and nursing are three concepts that articulate and complement each other. One cannot discuss health and nursing without including communication, so professionals must use appropriate communication to offer quality care^(28-29)^.

It was observed that services with organizational aspects, such as dedicated beds for fetal losses, institutional flow organization, and adequate staffing and preparation of professional teams, provide holistic and individualized care^(19)^. Women diagnosed with fetal death who witness other women with healthy children report a greater emotional imbalance, intensifying the feeling of sadness regarding the moment they are experiencing. It is emphasized that targeted care and empathetic communication help the family restructure after the loss^(18,21)^.

However, many healthcare professionals feel unable to explain the situation, either due to a lack of proper preparation or because of the pain and resentment the parents are dealing with. Communicating fetal death is one of the most delicate practices for the healthcare team, as it involves a moment laden with delicate emotions, requiring adequate and targeted training to deal with the reactions resulting from delivering bad news and all subsequent care^(15,25-26)^. For this reason, professionals working directly with these families need to understand the process to provide safe care to these families^(25,28-30)^.

Professionals, especially nurses, responsible for more comprehensive and direct care, sometimes distance themselves due to a lack of preparation, not knowing how to act in moments of loss^(23)^. In addition to support, families should be given time to address their questions, and if necessary, be referred to therapeutic groups and counseling with psychologists. Studies have shown that grieving parents experience more evident emotional pain after fetal loss when healthcare professionals cannot provide appropriate grief care^(27-30)^.

Training, as well as the education of nursing professionals on death and grief, was considered relevant to improve care for parents so that these professionals feel better prepared to handle this situation. Regarding training, studies suggest that services and their managers organize meetings and team gatherings to address the topic and preparation for dignified and holistic care that should be offered^(29-30)^.

Therefore, designing strategies to support family members is crucial. Nursing plays a diverse role in care, accompanying in a unique way, using effective communication techniques and therapeutic listening to try to prevent feelings such as guilt and the few memories of the child from being insufficient to overcome this delicate moment^(24-28)^.

Offering dignified care with the inclusion of the family group to understand aspirations and adapt decision-making, as well as providing ongoing education for all teams, not just nursing, on this topic, is of paramount importance. The uniqueness of each family must be respected, and their pains must be embraced and understood^(28-30)^.

### Study limitations

This study presents some limitations that should be considered when interpreting the results. Firstly, the selection of studies was restricted to the period from 2016 to 2021, with a significant concentration of studies in 2020. We also note a linguistic limitation, as eight out of nine selected studies were written in English, indicating low Brazilian production on the subject. Finally, we highlight the predominance of studies with evidence levels VI and V, emphasizing the need for more research based on robust evidence, such as randomized clinical trials and meta-analyses, to strengthen practical recommendations.

### Contributions to the Nursing and Health Field

Nursing care in situations of fetal death needs to be strengthened and supported by knowledge, adaptation, and sensitivity to families who have experienced loss and grief, contributing to a coping process permeated by support. In this perspective, professionals and services need to change the reality of the care offered to parents, promoting care specifically tailored to understanding the demands of families and building solid knowledge about the subject. This can foster a social, professional, and scientific movement that seeks to transform the reality of women’s, perinatal, and family health care.

The findings can serve as an incentive for the conduct of studies that individually address specific services and their nursing teams. Thus, they will highlight and deepen the topic, expanding knowledge and the appropriateness of care so that actions result in the valorization of loss and dignified assistance, as well as professional recognition for well-directed conduct.

In light of this, promoting training sessions and reflective moments that offer professionals opportunities for expression and attentive listening should be encouraged. Such actions facilitate support, ensuring that those engaged in this process can voice their anxieties, thereby enabling effective management in the aftermath of fetal death. It is believed that this research can prompt nursing professionals and healthcare services to reflect on the primary challenges associated with fetal loss and recognize the significance of effective communication in caregiving practices. This, in turn, can lead to the development of efficient care routines to enhance coping mechanisms with loss.

## FINAL CONSIDERATIONS

It is evident that there is a need to provide assistance in a differentiated, humanized, and scientifically grounded manner. Therefore, nurses need to contribute positively by participating in and conducting team training, providing the necessary information for holistic care to women and families. This care should not be limited to technical nursing aspects but should encompass the entire biopsychosocial context of parents and families experiencing fetal death.

The experience of grieving due to fetal death affects family dynamics, including the couple’s relationship, which can be strengthened when they face adversity together. In the social context, some of these parents suffer from a lack of recognition of their grief, the trivialization of their loss, and the refusal or delegitimization of mourning.

In this way, the contributions of this review emphasize the importance of qualified nursing care in the caregiving process for parents experiencing fetal death. Professional preparation makes a difference in this context; families should be welcomed, and their moments of anxieties, fears, and doubts respected to navigate grief in a less turbulent way, receiving support, empathy, and accurate information.
